# Health-related quality of life findings from the Faroe Islands FIT FIRST FOR ALL school-based physical activity study

**DOI:** 10.3389/fspor.2025.1533723

**Published:** 2025-06-25

**Authors:** Helgi Winther Olsen, Bára Berghamar Danielsen, Tórur Sjúrðarson, Søren Antoft, Peter Krustrup, Malte Nejst Larsen, Magni Mohr, Annika Helgadóttir Davidsen, May-Britt Skoradal

**Affiliations:** ^1^ Child Study Center, Faculty of Education, University of the Faroe Islands, Tórshavn, Faroe Islands; ^2^ Center of Health Science, Faculty of Health, University of the Faroe Islands, Tórshavn, Faroe Islands; ^3^ Department of Sports Science and Clinical Biomechanics, SDU Sport and Health Sciences Cluster (SHSC), Faculty of Health Sciences, University of Southern Denmark, Odense, Denmark; ^4^ Danish Institute for Advanced Study (DIAS), University of Southern Denmark, Odense, Denmark

**Keywords:** health-related quality of life (HRQOL), well-being, quality physical education, school-based physical activity, children and adolescents, school-wide intervention, FIT FIRST

## Abstract

**Introduction:**

This study evaluated the FIT FIRST FOR ALL program's effects on health-related quality of life (HRQOL) among Faroese schoolchildren aged 7–16 years during a 10-week school-wide intervention.

**Methods:**

A non-randomized controlled design included 360 pupils from an intervention (INT, *n* = 179) and a control school (CON, *n* = 181). The INT group participated in 3 weekly 40-minute physical activity sessions over 10 weeks. HRQOL was assessed using KIDSCREEN-52, and changes were analyzed using linear mixed-effects models.

**Results:**

A significant Time × Group interaction (*P* = 0.002) showed a 7% improvement in INT pupils' physical well-being, particularly among boys (8% increase, *P* < 0.001) and the youngest age group (7% increase, *P* = 0.006). No significant changes were observed in other HRQOL dimensions.

**Discussion:**

The FIT FIRST FOR ALL program improved physical well-being, particularly among boys and younger age groups, showcasing its potential to influence selected dimensions of HRQOL through structured school-based physical activity. These findings suggest that tailoring the program to specific age and gender groups could further strengthen its scalability and effectiveness across diverse school settings.

## 1 Introduction

Physical inactivity and sedentary behavior are growing global challenges, threatening the health and well-being of millions of children and adolescents (1, 2). Evidence shows that physical activity and sports participation are strongly linked to improved physical and mental health in young people (3, 4). Regular participation, particularly in team sports, boosts cardiovascular and musculoskeletal health and enhances physical and mental health (5). Despite these benefits, only 20% of children achieve the recommended 60 min of moderate-to-vigorous physical activity daily (6). To combat this, the World Health Organization aims to reduce global inactivity by 15% by 2030 (7).

Children spend a significant portion of their waking hours in school, making it an ideal setting to promote physical activity (8). Global physical activity policies identify schools as critical environments for integrating quality physical education as part of a whole-school approach, fostering health and physical literacy for lifelong active lifestyles and quality of life (1, 7, 9). However, despite this potential, many school-based interventions yield only modest improvements in physical fitness, body composition (10), and psychosocial outcomes (11), likely due to the complexities of their implementation and varied methodologies. Also, while interventions have been able to increase physical activity during school, they have not been found to increase overall daily physical activity (10, 12). The duration of the intervention—10 weeks—reflected what was feasible within the school schedule and aligns with previous studies showing that even longer interventions often produce only modest improvements in dimensions of health-related quality of life (HRQOL) (13), suggesting that factors beyond duration likely influence outcomes. This underscores the importance of intervention design, including how pupils experience participation. Current literature emphasizes the need for future studies to identify the most effective school-based physical activity interventions (6, 10, 14, 15). These studies should prioritize increasing physical activity before examining psychosocial outcomes and incorporate children's perspectives in their assessment (11).

To conceptually frame our focus on subjective outcomes related to HRQOL, we draw on Self-Determination Theory (SDT) (16), which posits that human well-being is supported when three basic psychological needs are fulfilled: autonomy, competence, and relatedness (16). In the context of school-based physical activity, these needs can be supported through inclusive, engaging, and appropriately challenging environments that promote enjoyment, effort, and social connection (17–20). The structure of FIT FIRST FOR ALL mirrors key features of Quality Physical Education (QPE): consistent session formats, age-appropriate design, full-class inclusion, and a non-evaluative atmosphere (21, 22). These design features are intended to support confidence, motivation, and perceived competence—particularly among pupils who may be less fit or less confident in typical PE settings (23–25). In this study, we assess HRQOL using the validated KIDSCREEN-52 instrument. Based on this framework, we expected the intervention to positively influence selected HRQOL dimensions, particularly those related to physical and psychological well-being. Age- and gender-related differences were also considered plausible, given prior findings showing that engagement and perceived benefit from school-based activity programs may vary across developmental stages and between boys and girls (24, 26). Where appropriate, we use the term well-being to refer specifically to the subscales Physical Well-being and Psychological Well-being (27, 28).

Physical Education (PE) is a mandatory component of the Faroese public school system, Fólkaskúli. This emphasis on physical activity highlights its integral role in the curriculum, creating an ideal framework for promoting quality physical education and implementing physical activity interventions. The system is modeled after the Danish Folkeskole and serves children from ages 7–16, with an optional grade 10 at age 17. Compulsory schooling includes three levels of three grades each: Level I (grades 1–3), Level II (grades 4–6), and Level III (grades 7–9). After completing these levels, most students advance to upper secondary or vocational training. PE is a required component of the curriculum from grades 1–9, covering swimming, ball games, gymnastics, dance, athletics, and outdoor activities. Grade 1 students receive 30 h of PE annually, which increases to 60 h per year from grades 2–9, averaging 1–2 h per week. Although students are evaluated within the national grading system, PE classes involve no formal exams or physical assessments and are primarily taught by trained PE teachers.

Despite limited data on Faroese children's physical activity levels, existing evidence aligns with global trends, indicating that only 20% meet recommended activity guidelines (6, 29, 30). Recent studies highlight a decline in self-reported health and psychosocial functioning among Faroese schoolchildren, including a rise in bullying (31). Furthermore, at study found that 61% of schoolchildren reported positive physical well-being, which is comparable to findings from an identical study in Denmark. The same study revealed that participation in leisure-time physical activity was associated with enhanced physical well-being, though disparities persist, with benefits diminishing for girls and adolescents (29).

To address this, a village school with approximately 200 pupils launched a government-supported FIT FIRST FOR ALL initiative, a 3-year (2022–2025) program aimed at improving physical health and psychosocial outcomes through a school-wide extracurricular physical activity intervention. The program's initial evaluation showed improvements in cardiorespiratory fitness and body composition (30); the present study focuses on its effects on HRQOL. The intervention introduced three 40-minute sessions of extracurricular physical activity per week during school hours, utilizing the FIT FIRST concept. This innovative, school-based program features structured, high-intensity sessions rooted in playful inclusive activities inspired by over 20 sports themes (32). The sessions are tailored for three age-specific groups: FIT FIRST 10 (ages 7–9), FIT FIRST 20 (ages 10–12), and FIT FIRST TEEN (ages 13–16). Each session emphasizes enjoyment and participation over competition, employing simple rules and team-based games to create a supportive environment. Furthermore, the program promotes both physical development and social interaction by organizing pupils into small groups, thereby fostering teamwork and inclusivity (33, 34).

Although the FIT FIRST FOR ALL concept combines these age-specific programs, it had not been implemented across an entire school or systematically evaluated over a 10-week period prior to this study. This project represents a first step in exploring its broader impact on pupils' physical health and quality of life.

### 1.1 Aim of the study

Building on our previously published findings demonstrating significant improvements in key health markers such as cardiorespiratory fitness, agility, and body composition across all age groups (30), the present study investigates whether the FIT FIRST FOR ALL intervention also affects pupils' HRQOL. While physical fitness improvements have been established, the relationship between structured physical activity and subjective outcomes such as HRQOL remains less clear, particularly in real-world school settings.

This study focuses on selected HRQOL dimensions measured using the KIDSCREEN-52 instrument. Our primary hypothesis posited that the 10-week FIT FIRST FOR ALL intervention would improve HRQOL, particularly in the dimensions related to physical and psychological well-being, as these are most plausibly influenced by the program's emphasis on effort, enjoyment, and perceived competence. We further hypothesized that these effects might vary by age and gender, based on prior findings indicating developmental and gender-based differences in motivation, perceived competence, and response to physical activity programs.

## 2 Methods

### 2.1 Study design

This non-randomized controlled study, best characterized as a non-randomized cluster trial (35), examined the impact of the FIT FIRST FOR ALL physical activity program on health-related fitness and HRQOL among Faroese schoolchildren. The study design was determined by the participating schools: one school had already committed to implementing the FIT FIRST FOR ALL program, while a neighboring school agreed to serve as a control. Random allocation was therefore not possible. Conducted during the spring term (February to May 2023), the 10-week intervention period reflected the available implementation window and involved 30 extracurricular physical activity sessions implemented at an intervention school (INT) in the Faroe Islands. The control school (CON), of comparable size, demographics, and regional characteristics, followed its standard curriculum without additional physical activities.

Pre- and post-intervention assessments included measures of health-related fitness, as outlined in prior research (30), and HRQOL, evaluated using the KIDSCREEN instrument. Pupils were categorized into three age groups according to the Faroese school system: level I (grades 1–3; ages 7–9), level II (grades 4–6; ages 10–12), and level III (grades 7–9; ages 13–16). These levels align with the age-specific adaptations of the FIT FIRST program.

### 2.2 Participants

**Figure 1:**
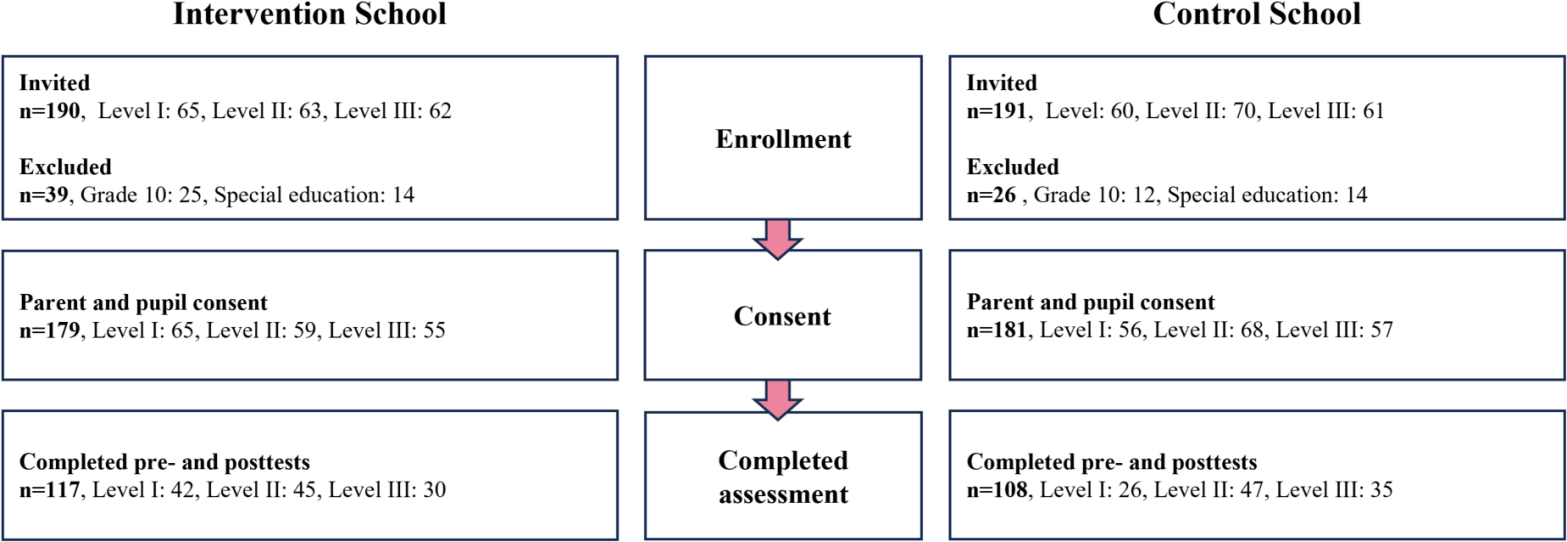
Flowchart comparing pupil participation in the FIT FIRST FOR ALL health-related quality of life (HRQOL) study. The figure shows the number of pupils invited, excluded, and included in the analytic sample with complete pre- and posttest HRQOL data, stratified by intervention and control school. Pupils in Grade 10 and in special education classes were excluded because their group sizes were too small for between-school comparisons.

Pupils aged 7–16 years from grades 1–9 were invited to participate (INT, *n* = 190; CON, *n* = 191). Pupils in grade 10 (INT, *n* = 25; CON, *n* = 12) and those enrolled in special education classes (INT = 14; CON = 14) were excluded from data collection. Children in special education classes were included in all teaching and PE sessions, but the small group size would not have allowed for meaningful analysis. For this reason, they were not invited to participate in fitness testing or questionnaire-based assessments. Of 381 eligible pupils, 365 received parental consent, with 360 pupils ultimately participating (INT, *n* = 179; CON, *n* = 181). Table 1 presents the baseline characteristics of pupils in each level, and a participation flowchart is seen in Figure 1.

**Table 1:** Participant characteristics at the time of pre-intervention tests.

Variable	Age group (lvl)	Intervention		Control	
		Mean ± SD	*n*	Mean ± SD	*n*
Age (yrs)	I	8.8 ± 0.9	65	8.7 ± 0.9	56
	II	11.8 ± 0.9	59	11.8 ± 0.9	68
	III	14.7 ± 1.0	55	14.6 ± 0.9	57
Height (cm)	I	134 ± 8	65	134 ± 8	56
	II	155 ± 9	59	155 ± 10	68
	III	168 ± 8	55	166 ± 8	57
Weight (kg)	I	34.2 ± 9.6	65	31.9 ± 7.9	56
	II	50.4 ± 13.3	59	50.1 ± 11.8	68
	III	64.6 ± 14.0	55	65.5 ± 17.9	57
BMI	I	18.7 ± 3.4	65	17.5 ± 2.7	56
	II	20.9 ± 4.3	59	20.6 ± 3.6	68
	III	22.7 ± 4.2	55	23.7 ± 5.5	57
Gender distribution [M/F (%)]	I	(52/48)	65	(41/59)	56
	II	(51/49)	59	(60/40)	68
	III	(45/55)	55	(53/47)	57
	ALL	(50/50)	179	(52/48)	181

BMI, body mass index; M, male; F, female; age group level I, 7–9 years; level II, 10–12 years; level III, 13–16 years.

### 2.3 Physical activity sessions

The FIT FIRST sessions were planned and conducted by trained PE teachers. Each class participated in 3 weekly 40-minute sessions over a 10-week period. Two PE teachers led each session with support from classroom teachers, supporting consistency and fostering active engagement. Teachers rotated FIT FIRST sports themes weekly, with each theme covering three sessions, resulting in a total of 30 sessions across the intervention period. All sessions were planned with the detailed age-related FIT FIRST programs (FIT FIRST 10, FIT FIRST 20, FIT FIRST TEEN). Teachers reported minor adjustments, such as slight modifications to game rules or activity duration to suit group size, facility availability, or pupil needs. Of the planned 3 weekly sessions, an average of 2.3 ± 0.2 sessions were completed for Level I, 2.2 ± 0.0 for Level II, and 2.1 ± 0.1 for Level III. Student attendance rates for the completed sessions were 84 ± 11% for Level I, 87 ± 11% for Level II, and 75 ± 24% for Level III.

### 2.4 Health-related quality of life (HRQOL) assessment

HRQOL was assessed using the KIDSCREEN-52, a validated self-report instrument designed to capture children's well-being across physical, psychological, social, family, and school domains (28). Its multidimensional structure reflects the WHO definition of health and incorporates child perspectives in line with current recommendations for school-based health research (27, 28). The tool includes 52 items divided across ten dimensions: *Physical well-being* (5 items), *Psychological well-being* (6 items), *Moods and emotions* (7 items), *Self-perception* (5 items), *Autonomy* (5 items), *Parent relations and home life* (6 items), *Financial resources* (3 items), *Peers and social support* (6 items), *School environment* (6 items) and *Social acceptance (bullying)* (3 items). Several of these domains—particularly physical and psychological well-being, autonomy, and social support—also reflect processes described in Self-Determination Theory, including perceived competence, social connection, and agency in health behaviors (16, 20, 24). Questions are answered on a 5-point Likert scale and transformed into *T*-values with a mean of 50 and standard deviation of 10 with higher scores indicating higher HRQOL. The KIDSCREEN-52 is appropriate for children and adolescents aged 8–18 and demonstrates good psychometric properties, including good internal consistency (Cronbach's *α*: 0.77–0.89) and test-retest reliability (ICC: 0.56–0.77) (27).

To ensure cultural relevance, the questionnaire was translated into Faroese by two native-speaking researchers. A professional translator then back translated it into English to confirm accuracy (36). This translation was tested on children aged 7–15 to verify comprehension and clarity.

### 2.5 Test administration

Testing took place in classrooms, where a trained researcher distributed the KIDSCREEN-52 questionnaire in paper form. Following a brief instructional introduction, the researcher read each question aloud while displaying it on a large screen for pupils to follow, ensuring accessibility for all literacy levels. The researcher remained neutral and addressed pupils' questions without influencing responses. Classroom teachers were also present and were briefed on their supportive role to avoid impact on pupil responses.

To accommodate attention spans, testing for grade 1 pupils was divided into two to three shorter sessions, totaling approximately 60 min. Pupils from grade 2 onwards completed the questionnaire within a single session of 30–40 min.

### 2.6 Analyses

Raw data was transcribed from paper and processed using R Statistical Software (version 4.4.1, R Core Team 2024), in line with KIDSCREEN guidelines (27). KIDSCREENS-52 item scores (1–5) were aggregated into ten dimensions, converted to Rasch person parameters and transformed into *T*-values. As defined in the KIDSCREEN manuals, single-item imputation was performed only when one item was missing within a given dimension. No further imputation procedures were applied; analyses were conducted using available cases.

Data analysis employed SPSS (version 30.0.0, IBM SPSS Statistics 2024) using linear mixed-effects models for repeated measures to evaluate outcomes (37). Statistical analyses were conducted based on the intention-to-treat (ITT) principle, whereby all participants were included in the analysis regardless of adherence to the intervention. However, only participants with complete pre- and post-intervention HRQOL data were included in the linear mixed-effects models, in line with a complete case approach. Three models addressed different study aspects, with statistical significance set at *P* < 0.05.

*A primary* analysis compared INT and CON groups with fixed effects for Time, Group, and their interaction (Time × Group) to assess the differences in response between the intervention school from pre- to post-intervention. Pairwise comparisons were performed for all endpoints to assess within-group differences across time points and between-group differences at baseline and post-intervention, with Sidak adjustment for multiple comparisons. The model was fitted using Restricted Maximum Likelihood (REML), and Kenward-Roger degrees of freedom were applied to obtain accurate standard error estimates. The repeated effect of Time was modeled for each subject, and appropriate covariance structures were used for both repeated measures and random intercepts. Residuals and predictions underwent graphical diagnostics.

*Secondary* sub-analyses were conducted to explore additional interactions for gender and for age group. A linear mixed model was employed with three-way interactions, incorporating fixed effects for Time, Group, Gender, and their interaction (Time × Group × Gender) to assess whether the Time × Group interaction differed by gender. Another sub-analysis included fixed effects for Time, Group, Level (age group), and their interaction (Time × Group × Level) to determine whether the Time × Group interaction varied across age groups. For both three-way interaction tests, Sidak-adjusted pairwise comparisons were conducted to assess within-group pre to post changes for gender and age group.

## 3 Results

### 3.1 Overall results

#### 3.1.1 Physical well-being

A significant Time × Group interaction effect was observed for Physical well-being (*P* = 0.002). INT showed an increase of 2.12 [0.68; 3.56] (*P* = 0.004), whereas CON exhibited a non-significant numerical reduction of −1.12 [−2.53; 0.30] (*P* = 0.12) (Table 2).

**Table 2:** KIDSCREEN *T*-value dimensional scores.

Intervention		Control		*P*
Pre	Post	Pre	Post	
Physical well-being				
46.1 [44.5; 47.8]^#^	48.2 [46.6; 49.9]**	48.9 [47.3; 50.5]	47.8 [46.2; 49.4]	0.002
Psychological well-being				
47.3 [45.6; 49.0]^#^	47.3 [45.6; 49.0]^#^	50.7 [49.1; 52.4]	51.4 [49.7; 53.1]	0.56
Moods and emotions				
45.1 [43.5; 46.7]	46.6 [44.0; 48.2]*^,#^	46.6 [45.1; 48.2]	49.5 [47.9; 51.1]**	0.28
Self-perception				
48.9 [47.3; 50.5]	50.0 [48.3; 51.6]^#^	49.5 [47.9; 51.1]	52.8 [51.2; 54.4]*	0.01
Autonomy				
46.4 [45.0; 47.8]	48.1 [46.6; 49.5]*	47.8 [46.4; 49.3]	49.5 [48.1; 51.0]*	0.98
Parent relations and home life				
47.4 [46.0; 48.9]	47.4 [45.9; 48.9]	48.0 [46.5; 49.4]	48.5 [47.0; 49.9]	0.55
Financial resources				
51.0 [49.5; 52.4]^#^	51.9 [50.5; 53.4]	53.0 [51.6; 54.5]	53.5 [52.1; 55.0]	0.61
Social support and peers				
46.7 [45.2; 48.3]^#^	46.5 [44.9; 48.1]	49.8 [48.3; 51.3]	48.5 [47.0; 50.1]	0.35
School environment				
46.0 [44.2; 47.8]^#^	45.7 [43.9; 47.6]^#^	51.1 [49.4; 52.9]	51.6 [49.8; 53.4]	0.54
Social acceptance (bullying)				
42.2 [40.4; 44.1]^#^	44.6 [42.7; 46.5]*^,#^	45.3 [43.4; 47.1]	47.8 [45.9; 49.6]*	0.91

Values are presented as mean KIDSCREEN *T*-value dimensional scores (with 95% confidence intervals) from a linear mixed model with Time, Group and Time × Group as explanatory variables. INT, *n* = 179 and CON, *n* = 181. The *P* interaction value (Time × Group) of the linear mixed model is presented. The result of the *post hoc* analysis is indicated by *P < 0.05, **P < 0.01 for within-group change from pre-intervention, and ^#^*P* < 0.05 compared with CON at the same timepoint. Parentheses () indicate a statistical tendency (*P* = 0.07).

#### 3.1.2 Psychological well-being

No significant intervention effect was detected for *Psychological well-being*. However, CON exhibited higher scores compared to INT both at pre- (*P* = 0.005) and post-intervention (*P* < 0.001) (Table 2).

#### 3.1.3 Moods and emotions

No significant between-group effect existed for *Emotional well-being*. Nevertheless, *post-hoc* analysis revealed a significant increase of 2.84 [1.19; 4.50] (*P* < 0.001) in CON and a borderline significant increase of 1.54 [−0.13; 3.22] (*P* = 0.07) in INT. As a result, CON had a significantly higher score compared to INT post-intervention, with a difference of 2.88 [0.59; 5.16] (*P* = 0.01) (Table 2).

#### 3.1.4 Self-perception

A significant Time × Group interaction effect was observed for *Self-perception* (*P* = 0.047). CON showed an increase of 3.32 [1.74; 4.90] (*P* < 0.001), while INT remained unchanged (1.06 [−0.53; 2.64], *P* = 0.19). This resulted in a significantly higher score in CON compared to INT post-intervention, with a difference of 2.84 [0.51; 5.17] (*P* = 0.02) (Table 2).

#### 3.1.5 Autonomy

No significant between-group effect was found for *Autonomy*. However, pairwise comparisons demonstrated significant increases in autonomy in both groups, with magnitudes of 1.71 [0.25; 3.17] (*P* = 0.02) in CON and 1.69 [0.22; 3.15] (*P* = 0.02) in INT (Table 2).

#### 3.1.6 Parent relations and home life

No significant between-group or within-group effects were observed for *Parent relations* (Table 2).

#### 3.1.7 Social support and peers

No intervention effect was observed for *Social support*. However, CON showed significantly higher scores compared to INT at pre-intervention (*P* = 0.006) (Table 2).

#### 3.1.8 Financial resources

No significant between-group or within-group effects were found for *Financial situation* (Table 2).

#### 3.1.9 School environment

No intervention effect was observed for *School environment*. However, CON had significantly higher scores compared to INT both at pre- and post-intervention (*P* < 0.001) (Table 2).

#### 3.1.10 Social acceptance (bullying)

No significant between-group effect existed for *Social acceptance (bullying)*. However, *post-hoc* analysis demonstrated significant increases in scores in both groups, with increases of 2.50 [0.61; 4.40] (*P* = 0.01) in CON and 2.35 [0.44;4.26] (*P* = 0.02) in INT (Table 2).

### 3.2 Sub-group/*post hoc* analyses

#### 3.2.1 The effect of gender

A significant three-way interaction between Time, Group, and Gender was observed for Physical well-being (*P* = 0.004), suggesting a gender-specific effect of the intervention on Physical well-being. *Post-hoc* analysis revealed that, in the intervention group, boys showed a marked improvement in Physical well-being score of 3.7 [1.7; 5.8] (*P* < 0.001), while girls exhibited no significant change (0.6 [−1.4; 2.6], *P* = 0.55). No within-gender changes were observed in the control group (Table 3).

**Table 3:** KIDSCREEN the effect of gender.

Gender	Intervention		Control		*P*
	Pre	Post	Pre	Post	
Physical well-being					
M	47.2 [44.9; 49.5]	50.9 [48.6; 53.2]***	49.7 [47.5; 51.9]	48.9 [46.6; 51.1]	0.004
F	45.0 [42.7; 47.3]	45.7 [43.3; 48.0]	48.1 [45.8; 50.4]	46.7 [44.3; 49.0]	
Psychological well-being					
M	48.0 [45.6; 50.3]	49.1 [46.7; 51.5]	51.6 [49.3; 51.1]	52.1 [50.0; 54.4]	0.70
F	46.6 [44.2; 49.1]	45.6 [43.2; 48.0]	49.8 [47.4; 52.2]	50.6 [48.2; 53.1]	
Moods and emotions					
M	45.9 [43.7; 48.1]	48.4 [46.1; 50.7]*	48.4 [46.2; 50.6]^#^	51.2 [49.0; 53.4]*	0.67
F	44.2 [41.9; 46.5]	44.9 [42.7; 47.2]	44.8 [42.5; 47.1]	47.6 [45.2; 49.9]*	
Self-perception					
M	50.7 [48.4; 52.9]	52.3 [50.0; 54.6]	50.5 [48.2; 52.7	54.8 [52.6; 57.1]***	0.17
F	47.1 [44.8; 52.9]	47.6 [45.3; 49.9]	48.4 [46.1; 50.7]	50.5 [48.1; 52.8]	
Autonomy					
M	48.6 [46.6; 50.5]	48.9 [46.9; 51.0]	48.9 [46.9; 50.9]	51.0 [49.1; 53.0]*	0.42
F	44.1 [42.1; 46.2]	47.2 [45.2; 49.2]**	46.7 [44.7; 48.7]	47.9 [45.8; 50.0]	
Parent relations and home life					
M	48.3 [46.3; 50.4]	48.8 [46.7; 50.9]	48.0 [46.0; 50.0]	49.3 [47.3; 51.3]	0.50
F	46.5 [44.4; 48.6]	45.9 [43.8; 48.0]	47.9 [45.8; 50.0]	47.5 [45.4; 49.7]	
Financial resources					
M	50.5 [48.4; 52.6]	51.8 [49.7; 53.9]	53.3 [51.3; 55.3]	54.8 [52.7; 56.8]	0.41
F	51.4 [49.3; 53.5]	52.1 [50.0; 54.2]	52.7 [50.6; 54.8]	52.2 [50.0; 54.3]	
Social support and peers					
M	46.8 [44.6; 49.0	46.7 [44.5; 49.0]	49.7 [47.5; 51.8]	48.5 [46.3; 50.6]	0.91
F	46.7 [44.4; 48.9]	46.3 [44.1; 48.5]	49.9 [47.7; 51.1]	48.6 [46.3; 50.9]	
School environment					
M	43.4 [41.0; 45.9]	44.5 [41.9; 47.0]	50.6 [48.2; 53.0]	50.8 [48.3; 53.2]	0.35
F	48.7 [46.1; 51.2]	47.1 [44.6; 49.7]	51.7 [49.2; 54.2]	52.5 [49.9; 55.1]	
Social acceptance (bullying)					
M	43.0 [40.5; 45.6]	44.0 [41.3; 46.6]	46.7 [44.1; 49.2]	48.7 [46.2; 51.2]	0.49
F	41.4 [38.7; 44.0]	45.2 [42.6; 47.8]**	43.7 [41.1; 46.4]	46.7 [44.0; 49.5]*	

Values are presented as mean KIDSCREEN *T*-value dimensional scores (with 95% confidence intervals) from a linear mixed model with time, group, gender and Time × Group × Gender as explanatory variables. INT, *n* = 179 (M, *n* = 89; F, *n* = 90) and CON, *n* = 181 (M, *n* = 94; F, *n* = 87). The *P* interaction value (Time × Group × Gender) of the linear mixed model is presented. The result of the *post hoc* analysis is indicated by *P < 0.05, **P < 0.01, ***P < 0.001 for within-gender change from pre-intervention. M, male; F, female.

No significant Time × Group × Gender interaction was found for any of the other variables measured. In general, *post-hoc* analyses revealed similar within-gender responses in both the intervention and control groups across other outcomes (Table 3).

#### 3.2.2 The effect of age-group

A significant three-way interaction between Time, Group, and Level was observed for Physical well-being (*P* = 0.02), School environment (*P* = 0.003) and Social acceptance (*P* = 0.004), suggesting an age-specific effect of the intervention on these psychosocial outcomes. For physical well-being, the *post-hoc* analysis revealed that, in the INT, only level I demonstrated significant improvement in Physical well-being score of 3.3 [0.9; 5.7] (*P* = 0.006), whereas in the CON, a borderline significant reduction was observed in level III of −2.5 [−4.9; 0.03] (*P* = 0.053) (Table 4).

**Table 4:** KIDSCREEN the effect of age group (level).

Level	Intervention		Control		*P*
	Pre	Post	Pre	Post	
Physical well-being					
I	50.8 [48.3; 53.3]	54.1 [51.6; 56.7]**	54.0 [51.4; 56.6]	53.2 [50.5; 55.9]	*P* = 0.02
II	43.6 [41.1; 46.2]	45.1 [42.5; 47.7]	48.5 [46.0; 50.9]	48.5 [46.0; 51.0]	
III	43.1 [40.3; 46.0]	44.5 [41.6; 47.3]	44.4 [41.7; 47.0]	41.9 [39.3; 44.6]*	
Psychological well-being					
I	52.2 [49.6; 54.8]	53.4 [50.7; 56.0]	55.5 [52.7; 58.2]	55.1 [52.2; 58.0]	*P* = 0.18
II	43.8 [41.1; 46.5]	43.2 [40.5; 46.0]	50.3 [47.7; 52.9]	52.3 [49.6; 54.9]	
III	45.2 [42.2; 48.2]	44.8 [41.8; 47.8]	46.3 [43.5; 49.1]	46.8 [44.0; 49.6]	
Moods and emotions					
I	45.4 [42.8; 48.1]	46.6 [43.9; 49.4]	46.8 [44.0; 49.6]	50.1 [47.1; 53.0]*	*P* = 0.42
II	43.0 [40.3; 45.7]	44.8 [42.1; 47.6]	47.7 [45.0; 50.3]	49.3 [46.6; 52.0]	
III	47.2 [44.1; 50.2]	48.7 [45.7; 51.7]	45.3 [42.4; 48.1]	49.1 [46.3; 51.9]*	
Self-perception					
I	53.0 [50.4; 55.5]	54.5 [51.9; 57.1]	54.2 [51.6; 56.9]	58.7 [55.9; 61.5]**	*P* = 0.11
II	47.3 [44.7; 49.9]	47.8 [45.1; 50.4]	50.7 [48.2; 53.2]	53.6 [51.0; 56.1]*	
III	45.6 [42.7; 48.5]	46.9 [44.0; 49.8]	43.3 [40.6; 46.0]	46.4 [43.7; 49.1]*	
Autonomy					
I	48.4 [46.1; 50.8]	48.9 [46.5; 51.3]	50.4 [47.9; 52.9]	51.3 [48.7; 53.9]	*P* = 0.44
II	45.6 [43.2; 48.1]	46.8 [44.3; 49.2]	47.4 [45.0; 49.7]	48.3 [45.9; 50.6]	
III	44.6 [41.9; 47.3]	48.6 [45.9; 51.3]**	45.8 [43.3; 48.3]	49.3 [46.8; 51.8]**	
Parent relations and home life					
I	48.9 [46.5; 51.3]	48.0 [45.6; 50.5]	50.3 [47.8; 52.8]	49.3 [46.7; 52.0]	*P* = 0.18
II	45.4 [42.9; 47.8]	45.9 [43.4; 48.4]	48.5 [46.1; 50.9]	49.4 [47.0; 51.8]	
III	48.1 [45.3; 50.9]	48.3 [45.5; 51.1]	44.9 [42.3; 47.4]	46.5 [44.0; 49.1]	
Financial resources					
I	48.3 [45.9; 50.6]	48.5 [46.1; 50.9]	50.6 [48.1; 53.1]	51.3 [48.8; 53.9]	*P* = 0.98
II	50.7 [48.3; 53.2]	52.1 [49.6; 54.5]	52.3 [50.0; 54.6]	52.7 [50.4; 55.1]	
III	54.8 [52.1; 57.4]	56.1 [53.4; 58.8]	56.3 [53.8; 58.7]	56.6 [54.1; 59.1]	
Social support and peers					
I	47.9 [45.3; 50.4]	47.8 [45.2; 50.4]	52.6 [50.0; 55.3]	49.7 [46.9; 52.5]*	*P* = 0.57
II	45.6 [43.0; 48.3]	45.5 [42.8; 48.2]	49.8 [47.3; 52.4]	49.2 [46.6; 51.8]	
III	46.6 [43.7; 49.5]	46.1 [43.2; 49.0]	46.8 [44.0; 49.5]	46.6 [43.9; 59.4]	
School environment					
I	47.4 [44.5; 50.2]	47.7 [44.8; 50.6]	57.7 [54.7; 60.7]	57.1 [53.9; 60.2]	*P* = 0.003
II	44.1 [41.2; 47.0]	42.1 [39.2; 45.1]	50.5 [47.7; 53.3]	50.4 [47.6; 53.2]	
III	46.4 [43.2; 49.6]	47.6 [44.4; 50.8]	45.0 [42.0; 48.1]	47.5 [44.4; 50.5]*	
Social acceptance (bullying)					
I	38.4 [35.5; 41.3]	38.7 [35.7; 41.6]	42.7 [39.6; 45.8]	46.7 [43.4; 50.0]*	*P* = 0.004
II	40.6 [37.6; 43.6]	45.2 [42.1; 48.2]**	47.7 [44.8; 50.5]	47.5 [44.6; 50.5]	
III	49.4 [46.0; 52.7]	51.2 [47.9; 54.5]	45.1 [41.9; 48.2]	49.1 [46.0; 52.2]*	

Values are presented as mean KIDSCREEN *T*-value dimensional scores (with 95% confidence intervals) from a linear mixed model with Time, Group, Level and Time × Group × Level as explanatory variables. INT, *n* = 179 (I, *n* = 65; II, *n* = 59; III, *n* = 55) and CON, *n* = 181 (I, *n* = 56; II, *n* = 68; III, *n* = 57). The *P* interaction value (Time × Group × Level) of the linear mixed model is presented. The result of the *post hoc* analysis is indicated by *P < 0.05, **P < 0.01 for within-age-group change from pre-intervention. Age groups Level I = 7–9 years; Level II = 10–12 years; Level III = 13–16 years.

For Social acceptance, the pairwise comparisons showed that, in INT, only level II demonstrated a significant increase of 4.6 [1.4; 7.8] (*P* = 0.005), whereas in CON, both level I and level III demonstrated significant improvements, with magnitudes of 4.0 [0.6; 7.5] (*P* = 0.02) and 4.0 [0.7; 7.3] (*P* = 0.02), respectively (Table 4).

## 4 Discussion

Our main finding of the HRQOL investigation is that the *Physical well-being* of 7–16-year-old school children improved following the 10-week FIT FIRST FOR ALL intervention, with the largest effects seen in boys and younger age groups. In contrast, the intervention had no measurable impact on other well-being domains, providing partial support for our primary hypothesis. Furthermore, we observed that the impact of the intervention was affected by gender and age, which is in line with our secondary and explorative hypothesis. While the study design did not allow for randomization, it followed the structure of a non-randomized cluster trial, which can yield valid comparative insights when contextual factors and baseline differences are acknowledged (35).

The choice of the KIDSCREEN-52 instrument, encompassing ten dimensions, reflects an understanding of HRQOL as a multifaceted construct of well-being (11, 28). While certain dimensions may be less relevant in the context of the Faroe Islands—where a school-based physical activity intervention is unlikely to influence them—the dimensions nonetheless offer value in evaluating its effectiveness and, in a sense, calibrating it to expected outcomes. For instance, scores for *Parent Relations* (47.4–48.5) and *Financial resources* (51.0–53.5) are near or above the European 50th percentile norm (27). These findings demonstrate that KIDSCREEN effectively captures the expected HRQOL of the population and aligns well with the anticipated scenarios anticipated by the schools' sociodemographic profiles (38). Some of the more responsive KIDSCREEN dimensions—particularly Physical well-being, autonomy, and social support—also align conceptually with motivational and social processes described in self-determination theory, including perceived competence, relatedness, and agency (20, 24).

Previous studies in the Faroe Islands have used a Danish translation of the shorter KIDSCREEN-27 instrument and found it effective for mapping HRQOL (39). However, this study marks the first time the KIDSCREEN-52 was translated into Faroese and implemented across an entire school population, which represents a significant methodological strength.

### 4.1 Physical well-being dimension

The primary outcome of the study was a significant overall increase in *Physical well-being*, reflected by a 4% improvement in INT, while CON experienced a non-significant 2% decrease. When comparing to European norm data (27), INT moved from a relatively low physical well-being ranked between the 25th and 50t percentile almost to the 50th percentile (49.63).

Interpretations should of course be careful, as school interventions are inherently complex. They take place within the dynamic environment of daily school life, making it challenging to blind and isolate specific effects and avoiding bias (10). While school-based physical activity interventions have yielded mixed results regarding physical fitness and well-being outcomes, research suggests that improvements in physical fitness are a prerequisite for enhancing well-being (11). This aligns with the findings of the current study, where significant increases in physical fitness and improved body composition among pupils in the intervention group (30) were accompanied by enhancements in their perceived physical well-being.

A notable strength of the current study is the structured FIT FIRST program and standardized manuals, which successfully facilitated these outcomes. The program incorporated intermittent high-intensity physical activity sessions designed to elicit elevated heart rates and significant musculoskeletal benefits, even for participants with minimal prior sports experience (33). Moreover, the sessions were led by skilled PE teachers specifically trained in the FIT FIRST methodology. Evidence highlights the pivotal role of specialist PE teachers, as their expertise, coupled with a well-designed curriculum, has a profound impact on pupils' fitness levels and overall physical activity (40, 41) and well-being (42). The program's consistent, developmentally appropriate, and inclusive structure reflects core principles of Quality Physical Education, and may have supported competence, motivation, and perceived inclusion—key psychological conditions for engagement, especially among pupils who are typically less confident or successful in PE (20, 24). Preliminary observations from a parallel, not-yet-published evaluation suggest that pupils with lower baseline fitness were equally engaged and reported enjoyment despite higher exertion, reinforcing the intervention's potential to support internal motivation and participation across subgroups. This delivery model—integrated into the school day and led by trained PE teachers—underscores the program's practical scalability and real-world applicability.

Interestingly, although fitness and body composition improvements were comparable across genders and age groups (30), the increase in physical well-being, as measured by the KIDSCREEN instrument, was predominantly driven by boys. Indeed, their scores increased significantly by 7% whereas no statistically significant change was detected for girls. By the conclusion of the intervention, boys approached the European 50th percentile (52.43), while girls remained within the 25th to 50th percentile range (42.53 and 47.08) (27). A potential explanation for the difference between boys and girls is gender-specific response bias in which girls and women are prone to report their well-being less positively than boys and men. This is apparent in several public health surveys where young women in particular report their mental and physical health lower than men (43). Research has also indicated that men tend to underreport symptoms of distress (44, 45).

Regarding age, solely the youngest group (level I) demonstrated a significant 6% improvement in physical well-being, surpassing the European 50th percentile (52.42) for 8–11-year-olds. Notably, both boys and the youngest age group, who drove the improvements, initially exhibited the highest physical well-being scores. In contrast, the older age groups (levels II and III) showed no significant changes, with scores ranking them between the 25th and 50th percentiles for 12–18-year-olds (42.53 and 47.08) (27).

This trend aligns with existing literature which highlights that populations most in need of well-being interventions—such as girls and adolescents—are often the most challenging to target (46). In contrast, an 11-week school-based physical activity intervention in the Faroe Islands targeting 10–12-year-olds did not yield comparable fitness improvements (26) but demonstrated significant enhancements in physical well-being, predominantly among girls (39). This outcome was likely driven by its emphasis on task-based games and health education within a multi-component framework, which aligns well with the preferences and engagement styles of this demographic (47, 48).

These findings should also be understood within the context of broad developmental and psychosocial variation. Children and adolescents aged 7–16 differ not only in physical and cognitive maturity, but also in motivational needs, perceptions of competence, and social sensitivity. Self-determination theory suggests that autonomy support and perceived competence are especially important during adolescence (20, 24), while younger pupils may benefit more from structure and play. In addition, gender norms may further influence engagement. Girls are more likely to report body-related discomfort or social self-consciousness in PE settings, while boys may disengage if they do not feel physically skilled or competitive (49, 50). These complexities highlight the difficulty of designing a single program that reaches all pupils equally.

Yet, the structure of FIT FIRST FOR ALL—non-evaluative, team-based, and predictably organized—creates conditions that support perceived safety, shared success, and motivation across subgroups. Preliminary observations from a parallel, not-yet-published evaluation suggest that even pupils with low baseline fitness—who are often harder to engage—reported enjoyment and effort acceptance. These observations align with findings that low-fitness pupils often feel unseen in traditional PE despite high effort (51), and that interventions without deliberate structural support tend to benefit those already doing well (46). Rather than tailoring to individual characteristics, scalable models may succeed by removing systemic barriers—particularly those embedded in competitive or performance-oriented PE environments. Programs that are flexible yet consistent, structured yet socially safe, may offer the best chance of reaching pupils with diverse developmental profiles.

These findings suggest either gender- and age-based differences in the time required for adaptation or the need for tailored adjustments, preferably through multicomponent approaches, to better support physical well-being improvements in girls and older pupils. A follow-up mixed-methods project has been initiated to examine pupil experiences and engagement through the lens of Self-Determination Theory, alongside quantitative evaluation of physical outcomes and implementation processes. Such research could investigate not only quantitative outcomes but also qualitative factors, including pupils' perceptions, teachers' strategies, and the broader school environment, to inform even more inclusive and effective intervention designs, as proposed by global policy stakeholders (8, 9). This focus on implementation is particularly relevant given that similar versions of the FIT FIRST program have shown divergent results across studies. While we previously reported positive changes among older pupils in cardiorespiratory fitness and body composition (30), other studies using the same FIT FIRST TEEN program have not found consistent physical effects in comparable adolescent samples (52). This contrast likely reflects differences in delivery context rather than program content. Supporting this, substantial variation in implementation fidelity and perceived feasibility has been observed across schools using the FIT FIRST 10 program (53). Teacher motivation, available facilities, and timetabling constraints all shaped how the program was enacted. These findings underscore that successful school-based physical activity interventions depend not only on design, but also on local ownership, alignment with school routines, and supportive environments.

### 4.2 Other HRQOL dimensions

The intervention's primary impact on physical well-being, the main outcome, offers several interpretations. First, the FIT FIRST FOR ALL program is specifically designed to improve participants' health-related fitness and physical well-being, which may explain the lack of significant (52, 53) effects on other HRQOL dimensions (9). While the frequency and duration of sessions, averaging 2.2 sessions per week each lasting 40 min, favor children's engagement and align with previous findings linking such activities to improved mental health outcomes (42), the intervention lasted only 10 weeks. It is likely that effects on other domains of HRQOL, such as autonomy, social support, or school environment, require longer or more socially focused interventions (19, 20). A promising initiative in Denmark extends the FIT FIRST concept over 2 years, incorporating strategies addressing diet, social media use, and sleep habits, which may provide valuable insights into its broader effects (54, 55).

Second, psychosocial outcomes like peer support or bullying can be understood as socially enacted phenomena, closely tied to group dynamics and the school environment as a whole (56). Compared to European KIDSCREEN norm data (27), INT pupils rated the dimensions *Social support and peers*, *School environment* and *Bullying* lower than the norm and lower than CON. As observed in neighboring countries, the incidence of bullying in Faroese primary schools has risen significantly over the past years (31). The low scores may, therefore, suggest broader challenges within the national school environment that could limit the intervention's effectiveness in psychosocial domains.

These outcomes can be interpreted in light of the program's design, which emphasizes exercise intensity and leverages group dynamics. Research consistently highlights the crucial role of enjoyment in fostering physical activity engagement and adherence among children and adolescents (57, 58). Immediate enjoyment, or state enjoyment, serves as a mediator for long-term intrinsic motivation, known as trait enjoyment (59). This immediate positive experience is significantly influenced by factors such as peer and group acceptance, positive feedback, and encouragement from teachers and classmates (50, 57). This reflects key motivational pathways described in self-determination theory, in which perceived social connection, autonomy, and competence enhance both momentary and lasting well-being outcomes (16, 20). The structured, inclusive design of the FIT FIRST FOR ALL sessions may have supported some of these needs—particularly competence and relatedness—offering a plausible link between the session experience and changes in HRQOL dimensions such as Physical well-being and social support (20). Although increasing intensity is generally associated with positive effects on children's and particularly adult's mental health (42), an interesting study exploring children's state enjoyment as a contextual construct of *attraction*, *preference*, *environment*, and *social factors* revealed a nuanced relationship between perceived enjoyment and exercise intensity (59). While children's preference for physical activity sessions generally increases with higher intensity, social and environmental factors often decline, potentially reducing perceived social enjoyment. This decline may result from a shift in group dynamics toward competitive individualism (59). This dynamic could help explain the observed improvements in the KIDSCREEN physical well-being dimension but the lack of measurable intervention-induced effects in the KIDSCREEN social domains.

A key strength and primary achievement of the FIT FIRST FOR ALL intervention was its success in increasing health-related physical fitness across the entire school (11, 30) by successfully introducing vigorous intensity sessions (15, 60). Previous studies have shown the FIT FIRST concept to generally be broadly inclusive and engaging while increasing fitness (33). However, while the program's success in fostering fitness gains is evident, its long-term effects on trait enjoyment and social HRQOL dimensions remain unclear, highlighting a limitation that future research should address. Pupils who do not feel recognized in typical PE settings may require more relational support to benefit emotionally (51), and sustained delivery by skilled teachers may be key to broader improvements in HRQOL (41). Finally, it is important to note that the subgroup analysis may be underpowered, and the results related to the explorative hypothesis should be interpreted with caution.

## 5 Conclusion

Our study showed a positive impact of structured physical activity on physical well-being among Faroese primary school pupils. The intervention did not significantly affect the other nine psychosocial dimensions measured with KIDSCREEN. This study contributes to a growing body of literature that calls for a multifaceted approach to school-based health interventions, emphasizing the need for sustained and culturally adapted programs that address both physical and psychosocial domains for a more holistic impact on children's HRQOL.

## Data Availability

The datasets presented in this article are not readily available because the datasets discussed in this article are not publicly available due to the sensitive nature of the information collected in a small-scale society, which could risk the identification of participants. While the research team is dedicated to fostering transparency and openness, safeguarding the privacy and rights of the participants takes precedence. Access to the datasets is therefore subject to strict protocols. Interested parties may contact the corresponding author for further information about potential access. Requests to access the datasets should be directed to helgio@setur.fo.
